# Odor tracking in aquatic organisms: the importance of temporal and spatial intermittency of the turbulent plume

**DOI:** 10.1038/s41598-020-64766-y

**Published:** 2020-05-14

**Authors:** Brenden T. Michaelis, Kyle W. Leathers, Yuriy V. Bobkov, Barry W. Ache, Jose C. Principe, Raheleh Baharloo, Il Memming Park, Matthew A. Reidenbach

**Affiliations:** 1 Department of Environmental Sciences, University of Virginia, Charlottesville, VA USA; 2 Department of Environmental Science, Policy, and Management, University of California Berkeley, Berkeley, CA USA; 3 Whitney Laboratory for Marine Bioscience, University of Florida, St. Augustine, FL USA; 4 Departments of Biology and Neuroscience, University of Florida, Gainesville, FL USA; 5 Department of Electrical and Computer Engineering, University of Florida, Gainesville, FL USA; 6 Department of Neurobiology and Behavior, Stony Brook University, Stony Brook, NY USA

**Keywords:** Olfactory system, Animal behaviour, Biomechanics, Marine biology

## Abstract

In aquatic and terrestrial environments, odorants are dispersed by currents that create concentration distributions that are spatially and temporally complex. Animals navigating in a plume must therefore rely upon intermittent, and time-varying information to find the source. Navigation has typically been studied as a spatial information problem, with the aim of movement towards higher mean concentrations. However, this spatial information alone, without information of the temporal dynamics of the plume, is insufficient to explain the accuracy and speed of many animals tracking odors. Recent studies have identified a subpopulation of olfactory receptor neurons (ORNs) that consist of intrinsically rhythmically active ‘bursting’ ORNs (bORNs) in the lobster, *Panulirus argus*. As a population, bORNs provide a neural mechanism dedicated to encoding the time between odor encounters. Using a numerical simulation of a large-scale plume, the lobster is used as a framework to construct a computer model to examine the utility of intermittency for orienting within a plume. Results show that plume intermittency is reliably detectable when sampling simulated odorants on the order of seconds, and provides the most information when animals search along the plume edge. Both the temporal and spatial variation in intermittency is predictably structured on scales relevant for a searching animal that encodes olfactory information utilizing bORNs, and therefore is suitable and useful as a navigational cue.

## Introduction

In the natural environment, animals must use intermittent, chemical cues to avoid predators, as well as find food and mates^1,2^. The dispersal of chemicals in both terrestrial and aquatic systems occurs through the advection of the fluid and by localized stirring caused by turbulent eddies^3,4^. The transport and mixing of odor cues therefore comprises a critical part of the sensory environment^5–8^. Previous experiments on dispersion of odorant plumes in both air and water have shown that the instantaneous concentration structure is highly filamentous, with high concentrations of odorant being surrounded by little or no odorant^9,10^. As these filaments are advected downstream, stretching and distortion of the filament occurs due to turbulence. This causes filament statistics to vary with time and distance from the source such that, on average, the plume becomes increasingly mixed and more homogeneous downstream^11^. Likewise, profiles of mean concentration in a transverse plane across a plume indicate a Gaussian distribution that widens with a decrease in centerline concentration with distance from the source. Although early odor tracking studies assumed that animals responded to these time-averaged concentration gradients in a plume^12^, it was later found that time-averaged concentrations converged too slowly to be useful to an animal while navigating in a plume^13^. In a similar fashion, it was also found that the sampling rates of many aquatic organisms were too slow to resolve the temporal rise in concentration within an odorant filament, and the spatial variation in the change in concentration too small to be reliably utilized in locating the source without long sampling periods^9,14^. Overall, the speed at which animal foraging occurs indicates that more instantaneous sensory feedbacks ae being utilized^10,15,16^.

Most research on olfaction in animals has been devoted to odor detection and discrimination based on highly sensitive detectors that respond to odorant concentration alone^17–21^. If the animal has excellent spatial memory and infinite time to roam the environment, it is likely that a large number of olfactory receptors could reconstruct a spatial map of the odor field or plume. However, the speed of maneuvers that these animals perform while they navigate within a plume suggests that more information is being processed^22,23^. Therefore, the question remains, how can these animals be so effective in their search strategies given the highly intermittent nature of odorants both in space and time?

### Search Strategies

Three of the most common established search strategies in organisms include chemotaxis, odor-gated rheotaxis, and infotaxis. Chemotaxis dictates following an increasing chemical concentration gradient, which changes in linear fashion within laminar flow^16^. Odor-gated rheotaxis combines mechanosensory information about water flow direction with chemosensing of chemical concentrations to inform movement^24^, i.e., if an organism detects odor, it will move in the upstream direction. Vergassola *et al*. introduced the concept of infotaxis, a search strategy for turbulent flow based on the fastest predicted acquisition of information^25^. Besides magnitude, a search variable is composed of spatial and temporal data, so a variable that includes both will be effective in infotaxis.

Odorants in aquatic environments, in particular oceans and rivers, move in intermittent and filamentous plumes, caused by the turbulent motion of the fluid. The challenges to studying chemosensory guided search are quantitatively understanding chemical transport and how organisms filter information to track an odor^24^. The temporal and spatial distributions of odor are thus complex^11,26^ and a proper assessment of the parameters used by animals actively undergoing search first requires an understanding of the dynamic odor landscape. This is apparent in the case of crustaceans, and lobsters in particular, which are model aquatic organisms for olfactory search.

### Lobster chemoreception and tracking

Lobsters utilize a multitude of sensors to inform search, composed primarily of sensory hairs called sensilla. Sensilla possess chemosensory cells, mechanosensory cells or bimodal receptors that contain both types of cells^6^. Chemosensory cells detect chemical concentrations and mechanosensory cells detect flow magnitude and direction. Although sensilla are located over the lobster’s entire body, aesthetasc sensilla are highly concentrated on the antennules. Crustaceans flick antennules to manipulate their fluid environment and enhance odorant sampling. Antennules flick down swiftly and rise slowly to lower the fluid Reynolds number and trap odorant laden water between their chemosensory hairs, called aesthetascs. This allows diffusive transport of odorant to chemosensors^27,28^. The effect is that animals can take discrete samples in time of the chemical environment with each flick^29^, and may be able to distinguish spatial information of odorants along the antennule length^9^. Inherent in the plume structure is information that can be utilized by the organism to estimate the relative distance to the source of odor^30–32^. From a biological standpoint, such dynamic segregation of sensory cues requires a neural mechanism for the estimation of intermittency timing that is usually considered to be associated with higher-order brain function and memory. Research suggests primary olfactory receptor neurons (ORN) can show two different patterns of activity. While the majority of ORNs are canonical, tonically-active ORNs that respond to concentrations of odorants with changes in discharge frequency, some are intrinsically or conditionally rhythmically active ORNs and referred to as ‘bursting’ ORNs (bORNs). bORNs have been identified across a range of taxa^33–40^, and have been well characterized in the olfactory organ of the spiny lobster, *Panulirus argus*, where they show intrinsic bursts in response to odorants^41^. bORNs respond to odorants in a phase-dependent manner; i.e., their response depends not just on the concentration of the odorant, but also on when the odorant arrives relative to their inherent bursting cycle. Each bORN in a population bursts at a different inherent frequency and is reset or “entrained” to burst based on the time of arrival of the odorant. Different odorant encounter intervals entrain different subsets of bORNS that, as a population, encode a range of time intervals that range between hundreds of milliseconds to multiple seconds. This dynamic encoding has been shown to hold across a broad range of odorant concentrations, and therefore bORNS have the capacity to accurately encode time intervals between odor encounters^42^. Indeed, a computational neural model for an ensemble of bORNs supports this hypothesis^7,42^. bORNs have been observed to burst probabilistically in response to stimulus depending on the cycle time of the cells^41^.

Canonical ORNs are known to respond to concentrations within this discrete sample, however, without memory of the previous time-course of odorant arrival it is currently unknown if canonical ORNs can be used to extract temporal variability of the plume. bORNs it appears, can quantify temporal aspects of the plume without higher-order brain function or memory of the last odorant encounter. Temporal olfactory information obtained from bORNs has several advantages over purely spatial data. The frequency of encountering odor in a plume increases with lateral proximity to the centerline directly downstream of the source and decreases farther downstream of the centerline^9^. Instantaneous data is often misleading however, as turbulence results in a chaotic distribution of high and low concentration odor filaments. Infotaxis and stimulus intensity-based decisions are promising as a search strategy, supported by many computational studies and robotic models in air and water, but fail to explain many movements observed in organisms and fall short to match animals searching performance^42–45^.

### Quantifying turbulent chemical plumes

The instantaneous temporal and spatial distribution of odors depends on factors such as distance from the source, the intensity of turbulence in the water flow and the topography over which the fluid is moving^46,47^. Techniques are required to quantify the movement and dispersal of odorants on the temporal and spatial scale at which an animal moves and samples their odorant environment. While laboratory methods, such as Particle Image Velocimetry (PIV) and Planar Laser Induced Fluorescence (PLIF), measure velocity and chemical concentration at adequate temporal and spatial resolution, the methods are often limited to two dimensional planes and/or fixed locations within a plume^48,49^. Although previous flume measurements have utilized these techniques to quantify the turbulent velocities and the filamentous structure of odorants within a plume^9^, they are not spatially or temporally coherent, meaning that they only provide snapshots of what a plume looks like at different points in time. An animal however, is continually moving and sampling its odor environment, and therefore it is necessary to be able to quantify plume structure at any location at any time instantaneously within a plume. Computational fluid dynamics (CFD) provides a technique to model three-dimensional flow at sufficient scales to resolve plume intermittency on the scale relevant to a lobster undergoing odor source search.

The specific questions addressed in this study are: (1) What is the spatial and temporal structure of plume intermittency in a turbulent odor landscape? (2) What are the search implications for perceiving plume structure through intermittency encoding by animals? To address these questions, we utilize simultaneous measurements of water velocities and odorant concentrations over a sand bed roughness within a large laboratory flume, as well as numerically simulate a similar turbulent plume using a 3-dimensional computational fluid dynamics model.

## Materials and Methods

### Computational model

**Figure 1:**
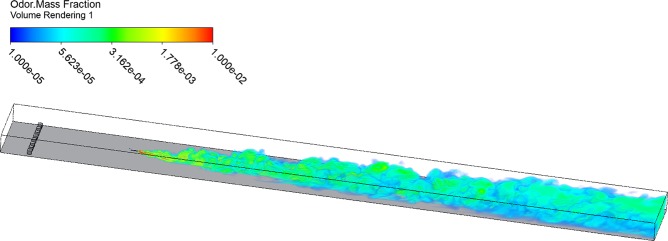
Simulated plume at t = 10 s. Flume section is 5 m long, 0.5 m wide, and 0.15 m tall. Odorant is isokinetically injected 1 m downstream from the water inlet. 3 cm roughness elements are placed 20 cm downstream of the inlet to trip the boundary layer. Flow is permitted in and out of the sides and top of the test section. Image was created using ANSYS R19 (ansys.com).

A hydrodynamic model was constructed for a unidirectional flow of sea water along a flat sandy bed in the CFX modeling package of ANSYS. In order to model the small-scale turbulence that can determine plume intermittency, the flow was modeled with the Smagorinsky Large Eddy Simulation (LES) model because of its ability to resolve individual turbulent eddies. The dimensions of the simulated section measured 5 m long, 0.5 m wide, and a depth of 0.15 m, Fig. 1. In order to generate a well-developed boundary layer and minimize the effects of side walls, flow in and out of the test section was allowed in the lateral directions and vertically at the top of the domain. The inlet boundary condition was a forced logarithmic boundary profile with a depth-averaged mean velocity of 2 × 10^−2^ m s^−1^. The characteristic roughness of the bed was set to 1 × 10^−3^ m consistent with a coarse sand roughness. A set of 3 cm cubes were placed near the inlet to trip the turbulent boundary layer. A simulated scalar odorant is injected isokinetically with the flow, as approximated by the inlet logarithmic boundary layer profile, at 3 × 10^−2^ m above the bed and 1 m downstream of the inlet to allow for turbulence to form before encountering the odorant. The odorant is specified to be neutrally buoyant with a diffusion coefficient in water of 1 × 10^−9^ m^2^ s^−1^, consistent with amino acids which commonly compose dissolved odors^50^. The fluid domain was simulated with approximately 20 million tetrahedral elements ranging from a size of 1.2 × 10^−4^ m near the boundaries and odorant inlet up to a maximum of 1 × 10^−3^ m, with an inflation layer along the bottom. The Kolmogorov length scale for the flow is 3.76 × 10^−4^ m. The model approximates sub-grid cell turbulence with eddy viscosity. The LES model was solved at a 2.5 × 10^−2^ s timestep for computational stability, but data were only recorded every five timesteps (Δt = 1 × 10^−1^ s). Recorded concentrations were normalized as a fraction of the source concentration, C_0_. The simulation was initialized by simulating 240 s before recording data, in order to record a developed plume.

### Validation of computational model

The computational model was validated using a physical seawater flume measuring 4 m long, 0.5 m wide, and 0.15 m deep. The flume was located at the Whitney Laboratory for Marine Bioscience at the University of Florida. The bed of the flume was a flat bed of coquina sand collected from a beach in Marineland, Florida. The sand was sieved and washed to remove particles smaller than 5 × 10^−3^ m in order to maintain optical clarity of the water in the flume (d_84_ = 8 × 10^−4^ m). Seawater was supplied by an inlet pipe, then the water was sent through a collimator at approximately 2 × 10^−2^ m s^−1^. Velocity measurements were made using Particle Image Velocimetry (PIV) while the plume structure was measured by coordinated Planar Laser Induced Fluorescence (PLIF). Fluorescein dye was injected isokinetically 3 × 10^−2^ m above the bed as a scalar tracer.

For the PLIF apparatus, a 473 nm laser was swept in a horizontal plane 3 × 10^−2^ m above the bed using a scanning moving-magnet mirror. The frequency of laser light is within the absorption peak of fluorescein (mean excitation at 490 nm) and the excited dye emits light at a mean wavelength of 520 nm^49^. The fluoresced dye was imaged using a 4 megapixel, 12 bit resolution, grayscale digital camera fitted with a 525 nm optical longpass filter. Four imaging fields of approximately 3 × 10^−1^ m by 1.5 × 10^−1^ m were placed around the centerline of the plume located 0.5 m, 1 m, 1.5 m, and 2 m downstream from the injection site. The injected concentration was increased while imaging locations further downstream in order to keep the emission intensity within the detectable range of the camera. All measured emission intensities were normalized by the source concentration, C_0_, during post-processing. The laser was scanned to illuminate the imaging field every 5 × 10^−2^ s (20 Hz). Each exposure and scan was 2.2 × 10^−2^ s long to reduce blurring of the moving odorant filaments. Raw images were processed to remove biases in the data, including varying pixel dark response, slow background changes in pH and temperature, lens and optics aberrations, and laser attenuation due to background concentrations^51^.

A second laser with an output wavelength of light at 532 nm was used for PIV. Since the 532 nm laser light was outside the absorption peak of fluorescein dye, the dye did not fluoresce. The laser was pulsed at 5 × 10^−2^ s intervals, alternating in time with the PLIF laser scanning. Particle locations illuminated by the PIV laser were recorded with the same camera as the PLIF images in alternating frames. The raw PIV images were modified in post processing using a method that caps pixel values to reduce overweighting of bright pixels^52^. The MatPIV 1.6.1 software package was used to calculate velocity vectors for a velocity resolution of 4.3 × 10^−3^ m, while concentration information was on the pixel scale, with a resolution of 1.3 × 10^−5^ m^53^.

Dye was injected isokinetically using a syringe pump through a 2 × 10^−3^ m inner diameter pipe 3 × 10^−2^ m above the bed. The molecular diffusion coefficient of fluorescein in water (1 × 10^−9^ m^2^ s^−1^) is within an order of magnitude to that of amino-acids (5 × 10^−10^ m^2^ s^−1^) which is an appropriate generalization for many odorants that aquatic organisms respond to. This makes the fluorescein plume an adequate representation for that of odorant encountered by an organism searching on a flat sand bed in a turbulence dominated fluid dynamic regime. 4800 sequential images were collected in each sampling run, equivalent to 120 s at a 40 Hz sampling rate. Between 3 and 5 runs were completed at 0.5 m, 1 m, 1.5 m, and 2 m downstream from the source, for a total of 15 runs. In all, 36,000 PIV images and 36,000 PLIF images were collected.

### Flow and concentration analysis

For the purpose of this analysis, intermittency is defined by the number of concentration spikes above the constant threshold concentration, C_thresh_, over a defined period of time. This definition is equivalent and more pragmatic than other studies who define intermittency as the proportion of time when the signal is absent^31^, because we utilize the measured variable, i.e. spike counts. We use a frequency definition for intermittency because it more directly represents how a bORN ensemble responds to a fluctuating odorant concentration. In addition, our definition of intermittency aligns more closely with other researchers who defined intermittency as the frequency or periodicity of odorant encounters^7,41,42^. The occurrence of a concentration spike is defined by a sequence of concentration samples where the first sample, C_(x,y,t)_, is below the threshold concentration, and the second, C_(x,y,t+1)_ is above the threshold concentration. Intermittency is inversely related to the time since the last concentration spike. Therefore, a portion of the plume said to have high intermittency is synonymous with saying that portion of the plume has very short time periods between threshold-crossing concentration spikes. Intermittency was calculated across the full 240 s, 10 Hz concentration timeseries at each simulated node at an elevation of 3 cm above the bed (n = 527,936). This method was repeated 5 more times except the temporal resolution of the concentration time series were limited to 5 Hz, 2 Hz, 1 Hz, 0.5 Hz, and 0.25 Hz by using every 2^nd^, 5^th^, 10^th^, 20^th^ and 40^th^ modeled concentration output, respectively. Intermittency was calculated for thresholds at every half order of magnitude ranging from C_thresh_ = C_0_x10^−7^ to C_0_x10^−3^ (i.e. C_0_x10^−7^, C_0_x10^−6.5^, C_0_x10^−6^, C_0_x10^−6.5^, …, C_0_x10^−3^).

### Search algorithms

Two search algorithms modeling odor tracking in crustaceans were simulated using 5,000 individual searches each. Start positions were randomly selected from a uniform distribution between 0.5 m and 3.5 m downstream from the source. Initial heading angles were randomly initialized from a uniform distribution between + /− 30° from upstream. Two antennules were modeled as two, point sensors whose positions were 5 cm away from the head + /− 30° from the heading angle. General guidelines for movement was to compare the two antennule readings, turn the heading angle 8° towards the stronger reading, and then move diagonally 30° from the heading angle towards the stronger reading. If both antennules have equal positive reading, then the algorithm moves forward along the heading angle. If both antennules have no reading, the algorithm waits. A movement speed of 5 × 10^−2^ m s^−1^ was used based on previous search algorithms in literature and observations of crustaceans^42^. The model makes a movement decision once every 0.5 seconds, consistent with a sampling frequency observed in *P. argus*^54^.

The first strategy used is a chemotropotactic search strategy. The reading at each antennule is the simulated odorant concentration at the point representing the antennule. When an antennule was not at a simulated node, a 2-dimensional linear interpolation between neighboring nodes was used to determine concentration. The second search algorithm incorporated intermittency of odorant encounters. Intermittency was determined for each antennule, defined as the number of odorant spikes, above a threshold concentration, encountered within the preceding 5 seconds. Odor detection within the previous five seconds was used to guide movement because the average spontaneous bursting frequency of bORNs is around 0.2 Hz^41,42^.

## Results

### Validation of computational model

**Figure 2:**
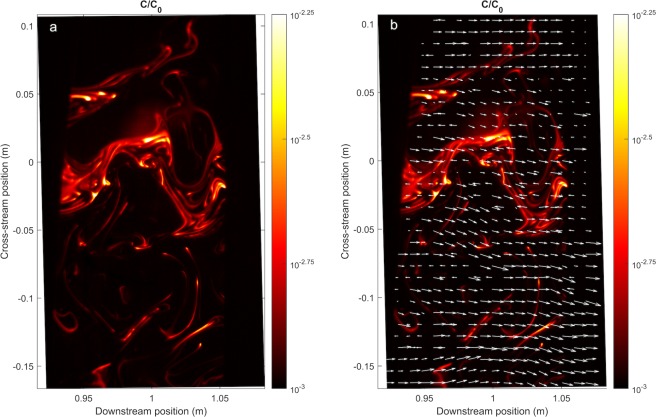
The filamentous and intermittent plume structure of the physical plume 1 m downstream from the source. **(a)** Normalized concentration 3 × 10^−2^ m above the bed; **(b)** Normalized concentration overlaid with a Galilean decomposition of local instantaneous velocities. A Galilean decomposition constant of 0.7 was used.

The concentration and velocity structure of an odorant plume was quantified within the 4 m long laboratory plume in order to validate the computational model. Utilizing alternating lasers, concentration was quantified using PLIF imaging, while velocity was quantified using PIV imaging, at an imaging rate of 40 Hz. Single frame images of the concentration and velocity structure of the plume at 3 cm above the bed and 1 m downstream from the source are shown in Fig. 2.

**Figure 3:**
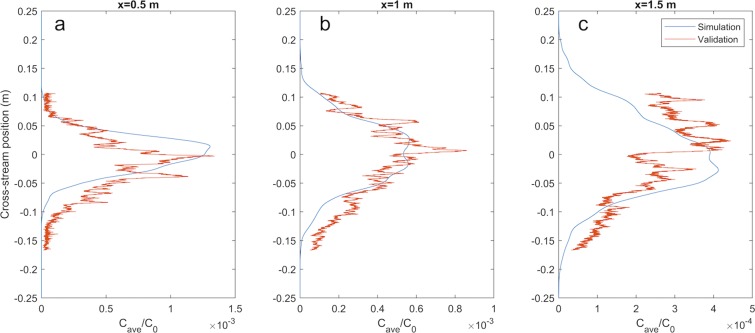
Comparison of simulated plume structure vs measured plume structure. The length of a fully developed turbulent plume was limited to about 1.5 m by the physical size of the flume used. The simulated plume dispersion sufficiently matches that of the physical plume for the purpose of this study.

The computational model was calibrated to recreate the concentration distribution of the physical plume created by flow over a sandy bed. The ultimate parameter set satisfactorily reproduced the time averaged concentration profiles at an elevation of 3 × 10^−2^ m above the bed, Fig. 3.

### Flow analysis

**Figure 4:**
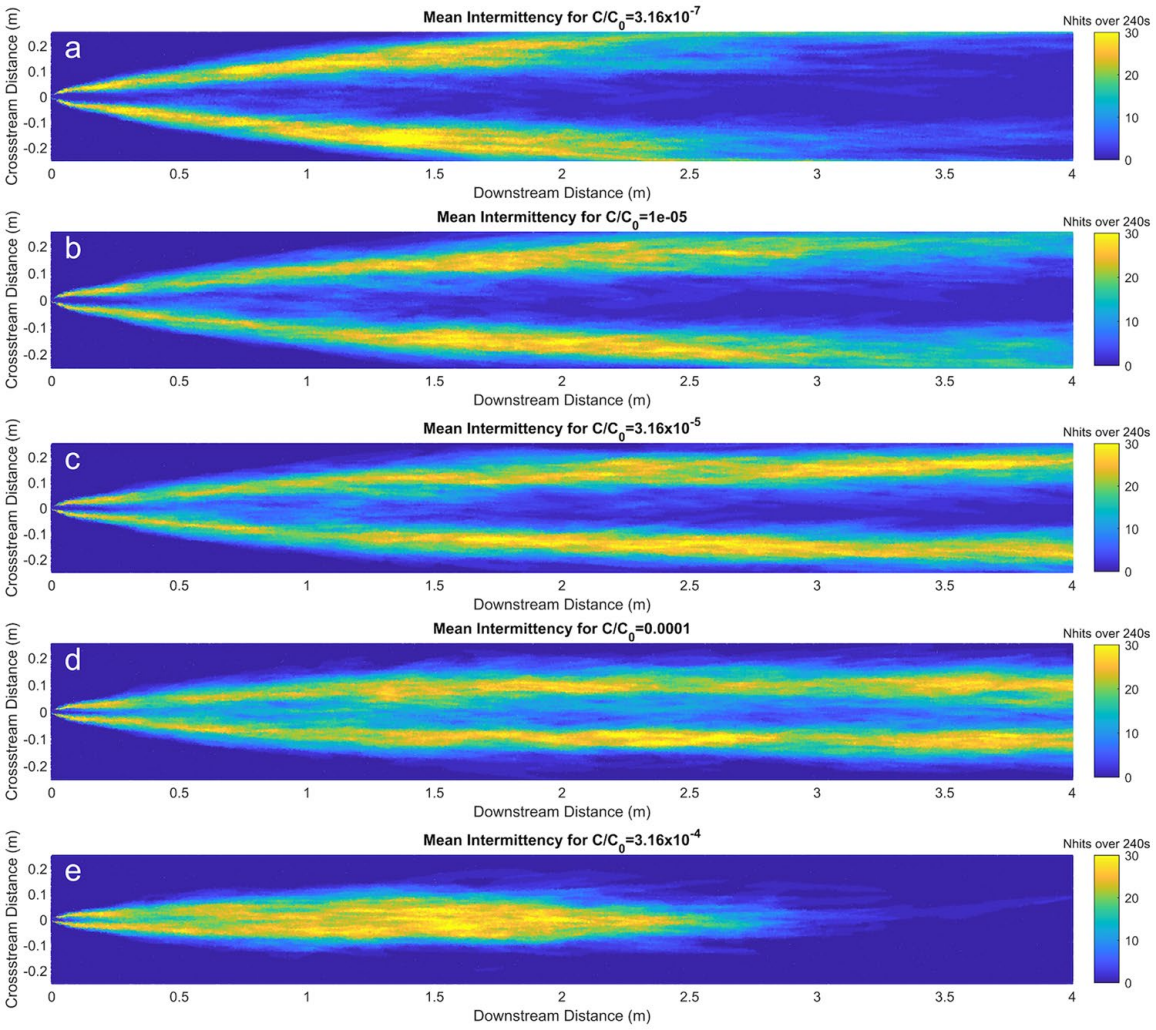
Number of instances the concentration time series for each simulated node on the z = 3 cm plane spiked above the threshold: **(a)** A low threshold concentration; **(b)** A threshold concentration half an order of magnitude smaller than the ideal threshold; **(c)** An ideal threshold concentration; **(d)** A threshold concentration half an order of magnitude larger than the ideal threshold; **(e)** A high threshold does not define the edge of the plume.

The odorant intermittency at 3 × 10^−2^ m above the bed over the full 240 s of simulation is displayed in Fig. 4 for five different threshold concentrations. The relationship was determined using the concentration time series recorded at 10 Hz at every simulated node 3 × 10^−2^ m above the bed. Predictably, the threshold concentration has a strong influence on the intermittency measured at a given point. A low threshold is seldom crossed around the centerline of the plume because the odorant concentration fluctuations are all well above the threshold. Also, predictably, a high threshold is rarely encountered on the edges of the plume. As seen in Fig. 4(c), the plume edge is well identified by intermittency when an intermediate threshold concentration of C_thresh_/C_0_ = 1 × 10^−4.5^ (3.16 × 10^−5^) is used. With this threshold, the plume edge is reliably more intermittent than inside and outside of the plume and the threshold is sufficient to delineate the edge of the plume over the length simulated. Therefore, this threshold concentration will be used for the subsequent analysis. It is important to note that similar spatial pattern for intermittency emerges when using threshold concentrations within half an order of magnitude of the ideal threshold.

**Figure 5:**
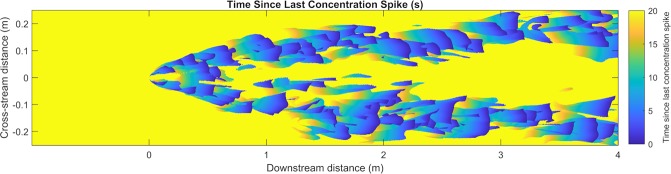
When looking at snapshot of time since the last concentration spike at t = 10 s, the edge of the plume is the most intermittent. C_thresh_/C_0_ = 3.16 × 10^−5^.

The spatial pattern of intermittency over the simulated 240 seconds is also consistent enough to be seen in an instantaneous snapshot of the time since the last concentration spike. As seen in Fig. 5, the time since the last concentration spike is reliably intermittent at the edge of the plume compared to inside or outside of the plume. Since bORNs respond to instantaneous odor stimuli in a phase-dependent manner^41^, this suggest that intermittency encoding shows the strongest response along the plume edge.

**Figure 6:**
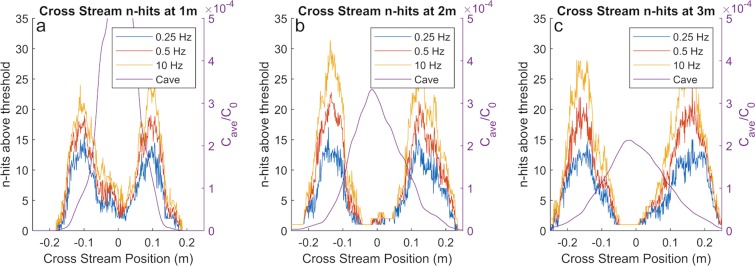
Three cross sections of number of concentration spikes detected at an elevation of 3 cm above the bed, at downstream positions (**a**) 1 m, (**b**) 2 m, and (**c**) 3 m, overlaid with time averaged concentration, C_ave_. The threshold concentration used to filter concentration spikes was C_thresh_/C_0_ = 3.16 × 10^−5^. Even at sampling frequencies of 0.25 Hz, elevated intermittency coincides with the plume edge. While faster sampling leads to a higher overall detected intermittency, it does not affect the cross-stream position of peak intermittency which can be utilized in a lobster while tracking the odorant plume.

**Figure 7:**
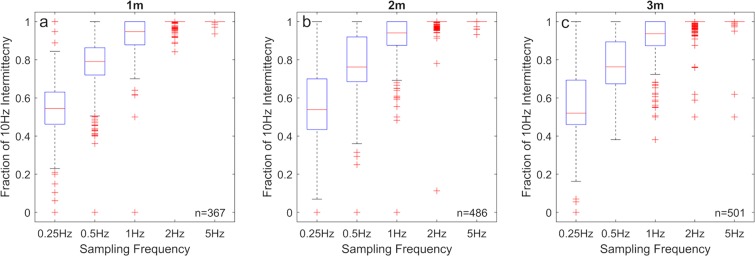
Boxplots comparing the number of detected concentration spikes for the three downstream locations of (**a**) 1 m, (**b**) 2 m, and (**c**) 3 m shown in Fig. 6. Each datapoint is the fraction of the number of detected concentration spikes measured at each simulated cross-stream position compared to the number of detected concentration spikes measured at 10 Hz. Positions where there were no concentration spikes observed at 10 Hz concentration sampling, were removed. n is the number of remaining simulated points in the cross section. Sampling concentration at 2 Hz and 5 Hz is shown to be effectively equivalent to 10 Hz for the purposes of observing concentration spikes.

Searching animals like *P. argus* are known to sample at frequencies slower than 10 Hz^54^. The edge of the plume, as characterized by elevated intermittency, is still prominent at concentration sampling rates (i.e. antennule flick rate) of 0.5 Hz and 0.25 Hz, Fig. 6. This supports that plume intermittency is detectable and provides useful information at the sampling frequencies that *P. argus* employs to sample the environment while searching^54^. The relationship of measured intermittency to sampling frequency is largely invariant over the length of the simulated plume. When sampling (i.e. flicking) at 1 Hz or 2 Hz, a lobster is able to capture most of the concentration spikes and therefore observe most of the intermittency. Essentially, the majority of concentration spikes in the plume are broad enough to be captured with a slower sampling rate of 1 Hz, Fig. 7.

### Application of intermittency to search

**Figure 8:**
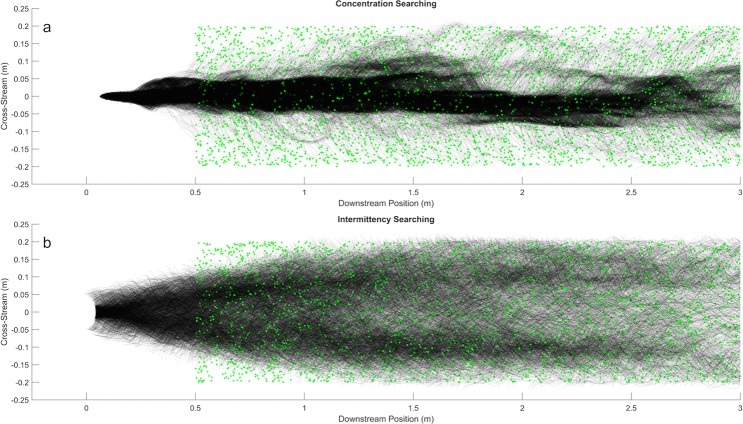
5000 searches randomly started between 0.5 m and 3.5 m downstream of the source. Each of the green dots is the random start locations for a search. Black lines show the trajectories of each search **(a)** Concentration gradient searchers path towards the centerline before navigating upstream; **(b)** Intermittency searchers preferentially follow the high intermittency at the edge of the plume.

To evaluate whether a searcher can effectively employ the intermittency information to find the source of odor, we used simple search algorithms (see Methods). As expected, the search algorithm incorporating intermittency of odorant encounters was able to dynamically track the plume edge in real time when moving at 5 × 10^−2^ m s^−1^. Despite sampling the plume at 0.5 Hz and only considering concentration spikes within 5 seconds, the intermittency algorithm searches were able to effectively navigate along the edge of the plume, Fig. 8. Conversely, a population of searches employing a concentration gradient search behavior, moving in the direction of highest measured concentration, distinctly navigated towards the centerline of the odorant plume.

## Discussion

The odor landscape for animals is a complex and dynamic regime where odorants are unevenly mixed with water over the length scales at which animals sample while navigating. Behavioral measurements have shown that this odorant variability is used as a directional cue^5,55,56^, suggesting the natural plume complexity includes useful information for navigating towards the source. Concentration intermittency, as detected by bORNs, is hypothesized to provide the directional cues that converge faster than time averaged concentration^42^. We show that the turbulence dynamics within a small-scale turbulent plume, mimicking a natural flow environment, generates intermittent concentration time series that have highest intermittency along either edge of the plume. With an appropriate threshold concentration, intermittency coincides with the plume edge but requires the organism to adapt to background concentrations of odorants. While the processes of setting the threshold sensitivity of the lobster tonically active ORNs are relatively well described^14^, little is known about the adaptation in bursting ORNs. Chemoreceptor cells undergo adaptation if an odor stimulus lasts too long, (i.e. their response to another odorant pulse of the same or lower concentration is reduced). This adaptation may be responsible for setting the threshold sensitivity of the neuron such that it can respond to pulses of odorant concentration higher than background^57^. Lobster ORNs also undergo cumulative adaptation (i.e. repeated odor pulses cause a gradual increase in latency time and decrease in number of spikes), which is more pronounced if there is a high rate of odor pulse delivery^58^. This adaptation allows lobsters to remain sensitive to transient increases in odorant concentration if the background concentration varies. Although understanding of mechanisms of adaptation in bORNs is beyond the scope of the current study, we hypothesize that the prolonged excitatory input would be potentially compensated to recover initial characteristics of a bORN rhythmic activity without affecting overall coding performance of the bORN ensemble.

The concentration spikes above a well calibrated threshold concentration that define the local intermittency are confined to a narrow cross-stream band at the edge of the plume. This suggests that intermittency can provide a robust and reliable indication of the edge of a turbulent plume which a searcher can utilize to navigate towards the source. While the ideal threshold concentration for this plume is not expected to be universal, there is precedence for neural adaptation to odor stimuli in *P. argus* lobsters^59,60^. Although adaptation timescales of antennule receptor neurons for *P. argus* are variable and depend upon a number of factors including concentration and flow conditions, it is reasonable to expect that the spatial relationship of intermittency along the plume edge will persist. More work will need to be done in order to identify the range of similar flow regimes that this result can be applied.

Plume intermittency is shown to be observable and predictable on time scales relevant for a searching animal on the order of seconds, as compared to the 10’s or 100’s of seconds required to establish a time averaged concentration gradient^16^. Additionally, the intermittency of the plume along its edge is effectively captured by an anatomically appropriate sampling rate for *P. argus* (1–2 Hz)^54^. Therefore, a lobster “sniffing” at a frequency typical of active searching, would be sampling the plume sufficiently to determine the intermittency and therefore be able to identify the edge of the plume. If an animal was employing an intermittency search strategy that aims to follow the edge of the plume, as opposed to a strategy that prioritizes following an instantaneous concentration gradient, we’d expect to see noticeably different search paths. Using very basic search algorithms, we show that a population of searches using intermittency directional cues tends to follow along the edge of the simulated turbulent plume while a population following concentration gradient cues tends towards the plume centerline. This edge searching behavior has been documented in *Callinectes sapidus* (blue crabs)^61^ and *Periplaneta Americana* (cockroaches)^62^. However, the behavior was attributed to comparing instantaneous concentration between inside the plume and outside of the plume using leg chemosensors^61^ or antennae^62^. Evidence for navigation using antennule information differential has been documented in *Homarus americanus* (American lobster)^5,8^. However, behavior was suggested to correspond to the slope of concentration peaks despite acknowledging that many concentration inputs do not result in turns, and some turns occur without concentration inputs. Intermittency poses a method by which the noisy concentration input is filtered and therefore could explain why all concentration peaks do not produce a direct behavioral response. Although the dynamic activity of tonically active ORNs is partially dependent on stimulus frequency and thus can contribute to the overall coding of temporal features of the signal, this response likely covers a different dynamical range than bORNS. Further analysis of the behavior of both tonically active ORNs and bORNs in parallel will allow for the establishment of the functional limits inherent in each of the two types of neurons.

The physiology of *P. argus* now appears to contain a neural mechanism for interpreting intermittency of odorant cues^4,7,29,42,63^. While it is unlikely that *P. argus* uses intermittency for navigation towards a source without incorporating other environmental and chemical cues, we establish the utility of high intermittency along the edge of an odorant plume within a natural turbulent boundary layer in a flow environment appropriate for *P. argus*. The average intermittency period along the edge is short enough to be relevant for the typical cycle time of bORNs and observed lobster search speeds. Flow dynamics suggest that an intermittency based search strategy should be distinguishable from a chemotaxis search strategy by having a propensity to follow the edge of a plume as opposed to the centerline. The average intermittency period is physiologically relevant at the observed search speeds of lobsters and within the range of stimulus intermittencies the ensemble of bORNs could encode. Animals, including lobsters, are known to contain a neural mechanism for interpreting intermittency of chemical cues^4,7,29,42,63^, now we show the plume dynamics for a typical boundary layer produces spatially unique and temporally predictable intermittency that can be used as an additional navigational cue to find the source of an odorant plume. It is expected that intermittency may play a fundamental role in the search for terrestrial organisms, although future studies will need to address intermittency within atmospheric plumes. We believe that incorporation of intermittency into search algorithms helps to construct a more accurate map of an odor landscape and can aid our understanding of animal navigation in turbulent odorant plumes.
